# COVID-19 in children and adolescents: MIS(-C)-taken diagnoses

**DOI:** 10.1007/s00431-022-04562-0

**Published:** 2022-07-21

**Authors:** M. van der Steen, P. L. Leroy, G. J. A. Driessen, M. A. G. E. Bannier

**Affiliations:** Department of Pediatrics, Maastricht University Medical Centre, Maastricht, The Netherlands

**Keywords:** COVID-19, Pediatrics, MIS-C

## Abstract

Multisystem inflammatory syndrome in children (MIS-C) is an inflammatory condition associated with severe acute respiratory syndrome coronavirus 2 (SARS-CoV-2). It is characterized by fever, gastro-intestinal symptoms, cardiovascular complications, conjunctivitis, skin involvement, elevated inflammatory markers, and coagulation abnormalities. The current ongoing COVID-19 pandemic causes an increased alertness to MIS-C. In combination with the heterogeneous clinical spectrum, this could potentially lead to diagnostic blindness, misdiagnosis of MIS-C, and overtreatment with expensive IVIG treatment. This report demonstrates the challenge of accurately distinguishing MIS-C from other more common inflammatory pediatric diseases, and the need to act with caution to avoid misdiagnoses in the current pandemic. We present a case series of 11 patients suspected of MIS-C based on the current definitions. Three of them were eventually diagnosed with a different disease.

*Conclusion*: Current definitions and diagnostic criteria lack specificity which potentially leads to misdiagnosis and overtreatment of MIS-C. We emphasize the need to act with caution in order to avoid MIS(-C)-taken diagnoses in the current pandemic.

**Table:** 

**What is Known:** *• A pediatric multisystem inflammatory disease associated with SARS-CoV-2 has been described (MIS-C).* *• There are three definitions being used for MIS-C, all including fever for at least 24 h, laboratory evidence of inflammation, clinically severe illness with multi-organ (≥ 2) involvement, and no alternative plausible diagnosis.*	
**What is New:** *• MIS-C has a heterogeneous clinical spectrum without distinctive features compared to more common childhood diseases. Current definitions and diagnostic criteria for MIS-C lack specificity which leads to misdiagnosis and overtreatment.* *• Amid the current excessive attention to COVID-19 and MIS-C, pediatricians should remain vigilant to avoid mistaken diagnoses.*

**Supplementary Information:**

The online version contains supplementary material available at 10.1007/s00431-022-04562-0.

## Introduction

Shortly after the first patients with severe acute respiratory syndrome coronavirus 2 (SARS-CoV-2) on December 2019, several countries reported young patients with a complex multisystem inflammatory disease associated with SARS-CoV-2. This disease is currently known as multisystem inflammatory syndrome in children (MIS-C).

MIS-C is characterized by fever, gastro-intestinal symptoms, cardiovascular complications, conjunctivitis, skin involvement, elevated inflammatory markers, and coagulation abnormalities [1]. Hemodynamic shock from either acute myocardial involvement or systemic hyperinflammation often requires intensive care admission with circulatory and respiratory support. Diagnostic testing frequently shows laboratory evidence of inflammation, coagulation disorders, increased cardiac biomarkers, and no indication of another causative diagnosis. The clinical spectrum and biological markers of MIS-C are heterogeneous and show overlap with other inflammatory conditions such as sepsis, Kawasaki disease, peritonitis, and toxic shock syndrome. Therefore, the clinical and biological features attributed to MIS-C lack the sensitivity and specificity to make a final diagnosis [1, 2]. Currently, three definitions are being used: MIS-C (described by US Centre of Disease Control (CDC)), PIMS-TS (pediatric inflammatory multisystem syndrome temporally associated with SARS-CoV-2, by the Royal College of Paediatrics and Child Health (RCPCH)), and multisystem inflammatory disorder in children and adolescents (WHO). All definitions include fever for at least 24 h, laboratory evidence of inflammation, clinically severe illness with multi-organ (≥ 2) involvement, and no alternative plausible diagnosis. The CDC and WHO definitions are more precise, requiring a proven association with SARS-CoV-2 by RT-PCR, serology, or antigen test; or by exposure to a suspected or confirmed COVID-19 case within 4 weeks prior to the onset of illness.

The similarity with Kawasaki’s disease has led to the recommendation to treat MIS-C patients with intravenous immunoglobulins (IVIG), whether or not in combination with acetyl salicylic acid and/or steroids, to prevent coronary aneurysmatic complications [3, 4].

The current ongoing COVID-19 pandemic causes an increased alertness to MIS-C. In combination with the heterogeneous clinical spectrum, this could potentially lead to diagnostic blindness, misdiagnosis of MIS-C, and overtreatment with expensive IVIG treatment. We present eleven patients admitted to our tertiary referral hospital because of severe illness and a high suspicion of MIS-C. In three of them, eventually, an alternative diagnosis was made. This report demonstrates the challenge of accurately distinguishing MIS-C from other more common inflammatory pediatric diseases, and the need to act with caution to avoid misdiagnoses in the current pandemic.

## Case descriptions

Between December 2020 and March 2021, eleven consecutive patients suspected of MIS-C were admitted to the pediatric ward of our tertiary referral hospital. Suspicion at presentation was based on clinical characteristics, laboratory, and/or echocardiographic findings, and a confirmed prior infection with COVID-19 or exposure to a suspected COVID-19 case within 4 weeks prior to the onset of illness. Three patients were eventually diagnosed with a different disease: pseudomonas sepsis with positive blood culture, inflammatory bowel disease (IBD), and perforated appendicitis based on imaging and surgery findings (non-MIS-C). Characteristics of non-MIS-C and MIS-C patients at presentation are summarized in respectively Table 1 and supplementary table. Ages of non-MIS-C patients were 15, 15, and 16 years. All MIS-C patients were aged < 12 years, including two children aged < 3 years (average 7.9 years). All patients had fever and gastro-intestinal symptoms. Tachycardia and/or hypotension was present in all non-MIS-C patients and 6/8 MIS-C patients. Conjunctivitis and/or skin rash was absent in non-MIS-C patients but present in 7/8 MIS-C patients. In all patients, laboratory evaluation showed inflammation with elevated CRP, ESR, ferritin, D-dimer, and fibrinogen. At presentation or shortly thereafter, NT-proBNP levels were elevated in all non-MIS-C patients and 7/8 MIS-C patients. Six patients, including one non-MIS-C patient with sepsis, had abnormal echocardiographic findings that are frequently seen in MIS-C, such as diastolic dysfunction, lowered shortening fraction, and broad coronary arteries. Because of the need for inotropics and respiratory support, four patients were admitted to the PICU including one non-MIS-C patient with sepsis.

**Table 1:** Characteristics at presentation of non-MIS-C patients

	**Patient 1**	**Patient 2**	**Patient 3**
**Characteristics**			
Age (yrs)	16	15	16
Gender	Female	Female	Male
**History/comorbidities**	Graves disease	None	None
	Tonsillitis 1 month before admission		
**Symptoms**			
No. of days of fever at presentation	5	2	5
Vomiting	Yes	No	Yes
Abdominal pain	Yes	Yes	No
Diarrhea	Yes	Yes	Yes
Tachycardia	Yes	Yes	Yes
Hypotension	Yes	Yes	Yes
Conjunctivitis	No	No	No
Skin rash	No	No	No
**Laboratory values**			
CRP (mg/L)	**229**	**249**	**123**
ESR (mm)	n/a	**43**	**43** (day 3)
Ferritin (mcg/L)	**294**	n/a	**570** (day 4) **(max 602)**
Leucocytes (/nL)	**0.1**	**14.2**	9.8
Lymphocytes (nL)	**0.1**	n/a	**0.6**
Hemoglobin (mmol/L)	7.8	7.4	9.1
Trombocytes (/nL)	280	**520**	162
D-dimers (mcg/L)	**911 (max 1707)**	6.2	**21,244** (day 3)
Fibrinogen (g/L)	**7.3**	**4.27**	**4.4** (day 3)
PT (sec)	**16.2**	14	n/a
aPTT (sec)	**37**	24	23 (day 2)
Troponin (ng/L)	**< 2.5 (max 83)**	< 3	17 (day 2)
NT-proBNP (pmol/L)	**5.6 (max 1015)**	**197**	**35** (day 2) **(max 295)**
SARS-CoV-2 RT-PCR	Negative	Negative	Negative
SARS-CoV-2 antibody	Negative	Negative	Negative
COVID-19 infection prior to presentation	No	No	No
Contact with COVID-19 positive subject	Yes	No	Possible
**Imaging**			
Echocardiogram	Shortening fraction 15–20%, diastolic dysfunction	No abnormalities	No abnormalities
**Fulfilled MIS-C criteria at presentation**			
WHO	Yes	No	Yes
CDC	Yes	No	Yes
RCPCH	Yes	Yes	Yes
**Treatment**			
Admission intensive care	Yes	No	No
Antibiotics	Yes	Yes	Yes
IVIG	Yes	No	Yes
Acetyl salicylic acid	Yes	No	Yes
Corticosteroids	No	No	Yes
Inotropics	Yes	No	No
Respiratory support	Yes (low flow)	No	No
**Eventual diagnosis**			
	Pseudomonas aeruginosa sepsis and typhlitis due to thiamazole-induced agranylocytosis	Inflammatory bowel disease	Perforated appendicitis

All patients were started on antibiotics at time of presentation because of the degree of illness. All MIS-C patients received IVIG. Five of them received high-dose corticosteroids because of ongoing inflammatory response after IVIG. All MIS-C patients were treated with prophylactic acetyl salicyl acid. One MIS-C patient had a thromboembolic complication for which she was treated with low-molecular-weight heparin. Two non-MIS-C patients were treated with IVIG and acetyl salicylic acid before eventual diagnosis was known (sepsis and perforated appendicitis).

All patients with non-MIS-C had a negative COVID-19 PCR and serology. Two of them had had contact with a COVID-19 positive subject several weeks prior to presentation. All MIS-C patients had positive COVID-19 serology. Based on clinical and diagnostic criteria, all non-MIS-C patients fulfilled the MIS-C definition according to the RCPCH, two patients met the criteria of the CDC and WHO definitions as well.

## Discussion

We describe eleven children presenting with symptoms resembling MIS-C, of whom three children eventually had a different diagnosis after MIS-C treatment had already been initiated. Our patients show the diagnostic challenges in distinguishing MIS-C from other pediatric inflammatory diseases during the COVID-19 pandemic due to the heterogeneous clinical and biochemical spectrum of MIS-C, and a probable expectation bias generated by the pandemic itself.

The average age of our MIS-C patients was 7.9 years, which is similar to previous findings [1] and lower compared to our non-MIS-C patients. Presence of gastro-intestinal or cardiovascular symptoms did not distinguish between non-MIS-C and MIS-C. In contrast, conjunctivitis and/or skin rash was only present in MIS-C patients, an association which has been reported before [5, 6]. However, not all MIS-C patients in our population had these symptoms, making it not a distinctive symptom. In our experience, multi-organ involvement, abnormalities on the echocardiogram, inflammatory, and hematologic markers did not differ substantially between non-MIS-C patients and MIS-C patients, in contrast to previous findings [2, 7–9].

Our case series highlight the non-specificity of the extensive laboratory evaluations as recommended in various MIS-C guidelines. These laboratory evaluations would probably also give abnormal results in various other infectious diseases but since these are not routinely performed, natural evolvement is unknown. The cardiac biomarkers troponin and NT-proBNP have received much attention regarding MIS-C. The first larger study on MIS-C showed elevated BNP levels in 73% of children with MIS-C [7]. This has triggered physicians to measure NT-proBNP more often in children presenting with symptoms resembling MIS-C. Elevated levels of NT-proBNP can be misleading in the diagnostic process, as shown in our three non-MIS-C patients who all had elevated NT-proBNP. The serum NT-proBNP concentration was first identified to be a marker for cardiac dysfunction. Myocyte stretch is the main stimulus for synthesis and secretion of proBNP from cardiac myocytes. ProBNP is thereafter split into the biologically active BNP and the inactive NT-proBNP. Later research demonstrated that serum NT-proBNP increases in sepsis patients and can be used to predict mortality in patients with sepsis [10]. In addition to mechanical factors, ischaemia, pro-inflammatory cytokines, such as tumor necrosis factor alpha and interleukin-1 beta, and neurohumoral factors including angiotensin II stimulate expression of BNP [11, 12]. Besides, NT-proBNP levels are also higher in case of hyperthyroidism, renal dysfunction, and when using certain medication [13]. The interpretation of NT-proBNP levels in children is complicated since the serum levels vary with age [14, 15]. Our findings in non-MIS-C patients confirm previous findings that NT-proBNP is also elevated in systemic inflammation and might not only have a cardiac source [16]. A recent meta-analysis has shown higher BNP levels in MIS-C patients vs. non-severe COVID-19 infections and in patients with severe MIS-C vs. non-severe MIS-C. Abnormal levels did not correlate with direct coronary artery lesions; however, cardiac markers may be monitored longitudinally in admitted MIS-C patients to predict potential deterioration during the disease course [17].

There is no definitive diagnostic test for MIS-C; therefore, a case definition for MIS-C has been formulated by the RCPCH, WHO and CDC, using broad criteria. At presentation, our non-MIS-C patients all met the RCPCH criteria for MIS-C and two met the WHO and CDC criteria as well based on (possible) contact with a COVID-19 positive subject, illustrating the non-specificity of these definitions. Eventually, PCR and serology for SARS-CoV-2 were negative in our non-MIS-C patients. It is questionable if diagnosing MIS-C without confirmation of a recent COVID-19 infection by positive PCR or serology is sufficient and justifies consecutive treatment. However, waiting for serology results generally takes time, potentially causing treatment delay. In MIS-C, this should be prevented as prompt treatment with IVIG is indicated. On the other hand, as the pandemic evolves, seropositivity for SARS-CoV-2 will become more common in children and it might no longer be indicative of a recent infection. This, and the possibility of vaccinating children, will make the use of serologic results in diagnosing MIS-C more complicated. It is, therefore, important to interpret serologic testing results in the context of the prevalence of viral transmission in the patient’s community and the probability of another causative disease. The latter stressing that a negative serologic test should prompt consideration of alternative diagnoses.

Treatment for MIS-C is based on stabilization of patients with shock and prevention of long-term cardiac consequences. There are currently no published results from randomized controlled clinical trials evaluating treatment options in MIS-C, but a multicenter open-label randomized-controlled platform study (RECOVERY) has been initiated to assess the short- and long-term outcomes of different treatments for MIS-C. Still, the American College of Rheumatology developed a clinical guidance for MIS-C based on experience in managing MIS-C, nonrandomized comparative cohort studies, and higher quality data from pediatric conditions with similar features [18]. Dual treatment with IVIG and glucocorticoids is associated with a lower risk of cardiovascular dysfunction and need for adjunctive immunomodulatory therapy on day 2, compared to IVIG alone, and is therefore considered as the first-line treatment. An important question is whether IVIG and corticosteroids have a potential deleterious impact on outcome in other diseases such as septic shock. Two non-MIS-C patients received IVIG and one corticosteroids as well, before actual diagnosis was made (sepsis and perforated appendicitis respectively). Studies on the use of corticosteroids during septic shock show conflicting results with some studies reporting adverse effects including a higher incidence of secondary infections and a higher mortality [19]. However, most studies show some potential in improving mortality rates and clinical markers. With respect to IVIG therapy, there is a risk of adverse effects in critically ill patients with severe infection, such as renal failure [20]. Despite some evidence that faster initiation of IVIG and glucocorticoids in MIS-C is associated with less admissions to the intensive care and a shorter hospital stay, patients under investigation for MIS-C without life-threatening symptoms should undergo a diagnostic evaluation for MIS-C as well as other possible causes to prevent the use of treatments that could be potentially harmful. Furthermore, IVIG is an expensive and scarce resource which should be taken into account when a diagnosis of MIS-C is questionable. All eleven patients received antibiotics at initial presentation because of the differential diagnosis of bacterial sepsis, a diagnosis which should not be missed since early detection and treatment have been shown to reduce mortality [21]. Therefore, blood cultures should be taken in patients suspected of MIS-C, preferably before any treatment is started.

The ongoing COVID-19 pandemic and increasing presence of MIS-C in media and scientific literature cause an increased alertness among physicians. This might create the illusion that MIS-C is highly prevalent among children and adolescents resulting in expectation bias, diagnostic blindness, and misdiagnosis. This was probably the case in the initial evaluation of our reported non-MIS-C patients. While evaluating a critically ill child, any exceptional circumstance such as the COVID-19 pandemic should not distract pediatricians from a thorough evaluation and focus on more common etiologies.

## Conclusion

Our case series demonstrates the difficulty in distinguishing MIS-C from other systemic diseases in children. Current diagnostic criteria and definitions lack specificity which potentially leads to misdiagnosis and overtreatment of MIS-C. Future studies are needed to better define MIS-C and the best therapeutic approach. Meanwhile, healthcare providers must remain vigilant to avoid MIS(-C)-taken diagnoses.

## Supplementary Information

Below is the link to the electronic supplementary material.

Supplementary file1 (PDF 442 kb)

## Abbreviations

BNP: Brain natriuretic peptide
CDC: US Centre of Disease Control
CRP: C-reactive protein
COVID: Coronavirus disease
ESR: Erythrocyte sedimentation rate
IBD: Inflammatory bowel disease
IVIG: Intravenous immunoglobulins
MIS-C: Multisystem Inflammatory Syndrome in Children
PICU: Pediatric intensive care unit
PIMS-TS: Paediatric Inflammatory Multisystem Syndrome Temporally associated with SARS-CoV-2
RCPCH: Royal College of Paediatrics and Child Health
RT-PCR: Reverse transcription polymerase chain reaction
SARS-CoV-2: Severe Acute Respiratory Syndrome Coronavirus 2
WHO: World Health Organization

## References

[1] Hoste L, Van Paemel R, Haerynck F (2021) Multisystem inflammatory syndrome in children related to COVID-19: a systematic review. Eur J Pediatr 10.1007/s00431-021-03993-5 PMC7890544 33599835

[2] Kelly MS, Fernandes ND, Carr AV, Lahoud-Rahme M, Cummings BM, Chiu JS (2021). Distinguishing features of patients evaluated for multisystem inflammatory syndrome in children. Pediatr Emerg Care.

[3] Jiang L, Tang K, Levin M, Irfan O, Morris SK, Wilson K (2020). COVID-19 and multisystem inflammatory syndrome in children and adolescents. Lancet Infect Dis.

[4] Ouldali N, Toubiana J, Antona D, Javouhey E, Madhi F, Lorrot M (2021). Association of intravenous immunoglobulins plus methylprednisolone vs immunoglobulins alone with course of fever in multisystem inflammatory syndrome in children. JAMA.

[5] Esposito S, Principi N (2021). Multisystem inflammatory syndrome in children related to SARS-CoV-2. Paediatr Drugs.

[6] Newburger JW, Takahashi M, Gerber MA, Gewitz MH, Tani LY, Burns JC (2004). Diagnosis, treatment, and long-term management of Kawasaki disease: a statement for health professionals from the Committee on Rheumatic Fever, Endocarditis, and Kawasaki Disease, Council on Cardiovascular Disease in the Young American Heart Association. Pediatrics.

[7] Feldstein LR, Rose EB, Horwitz SM, Collins JP, Newhams MM, Son MBF (2020). Multisystem inflammatory syndrome in U.S. children and adolescents. N Engl J Med.

[8] Aydin F, Celikel E, Ekici Tekin Z, Coskun S, Sezer M, Karagol C (2021). Comparison of baseline laboratory findings of macrophage activation syndrome complicating systemic juvenile idiopathic arthritis and multisystem inflammatory syndrome in children. Int J Rheum Dis.

[9] Feldstein LR, Tenforde MW, Friedman KG, Newhams M, Rose EB, Dapul H (2021). Characteristics and Outcomes of US children and adolescents with multisystem inflammatory syndrome in children (MIS-C) compared with severe acute COVID-19. JAMA.

[10] Sun YN, Lin CW, Tang W, Zhang JP (2021). Age-stratified cut-off values for serum levels of N-terminal proB-type natriuretic peptide and mortality from sepsis in children under age 18 years: a retrospective study from a single center. Med Sci Monit.

[11] Ma KK, Ogawa T, de Bold AJ (2004). Selective upregulation of cardiac brain natriuretic peptide at the transcriptional and translational levels by pro-inflammatory cytokines and by conditioned medium derived from mixed lymphocyte reactions via p38 MAP kinase. J Mol Cell Cardiol.

[12] Nishikimi T, Kuwahara K, Nakao K (2011). Current biochemistry, molecular biology, and clinical relevance of natriuretic peptides. J Cardiol.

[13] Nishikimi T, Nakagawa Y (2021). Potential pitfalls when interpreting plasma BNP levels in heart failure practice. J Cardiol.

[14] Rauh M, Koch A (2003). Plasma N-terminal pro-B-type natriuretic peptide concentrations in a control population of infants and children. Clin Chem.

[15] Nir A, Lindinger A, Rauh M, Bar-Oz B, Laer S, Schwachtgen L (2009). NT-pro-B-type natriuretic peptide in infants and children: reference values based on combined data from four studies. Pediatr Cardiol.

[16] Jensen J, Ma LP, Fu ML, Svaninger D, Lundberg PA, Hammarsten O (2010). Inflammation increases NT-proBNP and the NT-proBNP/BNP ratio. Clin Res Cardiol.

[17] Zhao Y, Patel J, Huang Y, Yin L, Tang L (2021). Cardiac markers of multisystem inflammatory syndrome in children (MIS-C) in COVID-19 patients: a meta-analysis. Am J Emerg Med.

[18] Henderson LA, Canna SW, Friedman KG, Gorelik M, Lapidus SK, Bassiri H et al (2022) American College of rheumatology clinical guidance for multisystem inflammatory syndrome in children associated with SARS-CoV-2 and Hyperinflammation in pediatric COVID-19: version 3. Arthritis Rheumatol 10.1002/art.42062 PMC9011620 35118829

[19] Pourmand A, Whiteside T, Yamane D, Rashed A, Mazer-Amirshahi M (2019). The controversial role of corticosteroids in septic shock. Am J Emerg Med.

[20] Aubron C, Berteau F, Sparrow RL (2019). Intravenous immunoglobulin for adjunctive treatment of severe infections in ICUs. Curr Opin Crit Care.

[21] Hilarius KWE, Skippen PW, Kissoon N (2020). Early recognition and emergency treatment of sepsis and septic shock in children. Pediatr Emerg Care.

