# Supplementary material for: COVID-19 in children and adolescents: MIS(-C)-taken diagnoses
Source: Eur J Pediatr. 2022 Jul 21;181(9):3549–54. doi: 10.1007/s00431-022-04562-0 (PMC9302213; doi:10.1007/s00431-022-04562-0)
Supplement: Supplementary file 1 — Supplementary file1 (PDF 442 kb) [file 431_2022_4562_MOESM1_ESM.pdf]

	Patient 1	Patient 2	Patient 3	Patient 4	Patient 5	Patient 6	Patient 7	Patient 8
**Characteristics**								
Age (yrs)	9	10	9	11	11	2	1	10
Gender	female	male	male	male	female	female	male	male
**History / Comorbidities**	None	COVID-19 infection 6 weeks prior to presentation	COVID-19 infection 4 weeks prior to presentation	None	None	COVID-19 infection 3-4 weeks prior to presentation, Eczema	None	COVID-19 infection 5 weeks prior to presentation
**Symptoms**								
No. of days of fever at presentation	6	6	6	6	7	6	5	5
Vomiting	yes	yes	yes	yes	yes	no	no	no
Abdominal pain	yes	yes	yes	yes	yes	yes	no	yes
Diarrhoea	no	yes	yes	yes	no	yes	yes	no
Tachycardia	yes	yes	yes	yes	yes	no	yes	no
Hypotension	yes	no	yes	yes	no	no	no	no
Conjunctivitis	no	no	yes	yes	yes	yes	yes	no
Skin rash	no	yes	no	yes	yes	yes	yes	yes
**Laboratory values**								
CRP (mg/L)	**148**	**70**	**244**	**250**	**147**	**127**	**57 (max 90)**	**88 (max 115)**
ESR (mm)	**41**	**30**	**50** (day 2)	n/a	**61** (day 3)	n/a	n/a	n/a
Ferritin (mcg/L)	**1249**	**499**	**4907**	**782**	**902** (day 4)	n/a	**330**	**231 (max 770)**
Leucocytes (/nL)	**24.4**	8.0	6.9	**17.7**	10.0 (day 3)	n/a	8.6	8.4
Lymphocytes (/nL)	**0.5**	**0.55**	**0.48**	**1.6**	2.1 (day 3)	n/a	**1.4**	n/a
Hemoglobin (mmol/L)	**6.6**	7.3	8.3	**6.1**	**6.3** (day 3)	**5.5**	**6.6**	8.2
Trombocytes (/nL)	**35**	**137**	**138**	305	199 (day 3)	205	243	176
D-dimers (mcg/L)	**8810** (day 2)	**4201**	**>10000**	**2400**	**987** (day 4)	**5810**	**5870 (max 8321)**	**2429 (max 2707)**
Fibrinogen (g/L)	**5.9**	**6.9**	**5.0**	**9.8**	**4.0** (day 4)	**5.5**	**5.2**	**5.4**
PT (sec)	**17.8**	11.3	**11.9**	n/a	11.3 (day 4)	11.2	**11.9**	12.2
aPTT (sec)	**47**	30	**39**	n/a	**39** (day 4)	30	30	27
Troponin (ng/L)	**227**	8 **(max 20)**	**210 (max 269)**	**42**	5 (day 3)	11	7	6
NT-proBNP (pmol/L)	**3542**	**85.8 (max 413)**	**687**	**2402**	**307**	**1058**	**591 (max 720)**	**103 (max 390)**
SARS-CoV-2 RT-PCR	negative	negative	positive	positive	positive	negative	negative	positive
SARS-CoV-2 antibody	positive	positive	positive	positive	positive	positive	positive	positive
COVID-19 infection prior to presentation	no	yes	yes	no	no	yes	no	yes
Contact with COVID-19 positive subject	yes	yes	yes	yes 3 weeks prior to presentation	yes	yes	yes 4 weeks prior to presentation	yes
**Imaging**								
Echocardiogram	diastolic dysfunction	no abnormalities	no abnormalities	diastolic dysfunction	no abnormalities	diastolic dysfunction	broad coronary artery	broad coronary artery
**Fullfilled MIS-C criteria at presentation**								
WHO	yes	yes	yes	yes	yes	yes	yes	yes
CDC	yes	yes	yes	yes	yes	yes	yes	yes
RCPCH	yes	yes	yes	yes	yes	yes	yes	yes
**Treatment**								
Admission intensive care	yes	no	no	yes	no	yes	no	no
Antibiotics	yes	yes	yes	yes	yes	yes	yes	yes
IVIG	yes	yes	yes	yes	yes	yes	yes	yes
Acetyl salicylic acid	yes	yes	yes	yes	no	yes	yes	yes
Corticosteroids	yes	yes	yes	no	no	yes	yes	no
Inotropics	yes	no	no	no	no	no	no	no
Respiratory support	yes	no	yes	yes	no	no	no	no
**Eventual diagnosis**								
	MIS-C	MIS-C	MIS-C	MIS-C	MIS-C	MIS-C	MIS-C	MIS-C with intracranial hypertension + appendicitis
